# Supplementary material for: Effect of community-led delivery of HIV self-testing on HIV testing and antiretroviral therapy initiation in Malawi: A cluster-randomised trial
Source: PLoS Med. 2021 May 11;18(5):e1003608. doi: 10.1371/journal.pmed.1003608 (PMC8112698; doi:10.1371/journal.pmed.1003608)
Supplement: S1 Text — Statistical analysis plan for primary and secondary outcomes. (DOCX) [file pmed.1003608.s002.docx]

**Community-led distribution of HIV self-tests: a cluster-randomised trial investigating uptake of HIV testing, linkage to treatment and prevention, costs and safety in rural Malawi (protocol version 2.1)**

**HIV Self-Testing AfRica (STAR) Initiative**

# Statistical analysis plan

This document outlines the statistical analysis plan for a cluster-randomised trial of community-led delivery of HIV self-testing kits (protocol version 2.1).

1. **Study design**
   1. **Study arms**

Thirty group village heads clusters were allocated using restricted 1:1 randomisation to either:

- **Community-led HIVST arm**: Community representatives are supported to plan and administer an HIVST campaign linked to HIV care and prevention in their communities. Specifically, community health action groups and government community health workers attend participatory workshops to plan the campaign. Community volunteers also receive an HIVST training. Community representatives implement the seven-day campaign, with HIVST kits (OraQuick HIV Self-Test), instructional materials, data collection tools, and gratuity provided.
- **SOC arm**: No HIVST kits are available. Across arms, HIV testing is provided at health facilities based on the national testing algorithm. “Treat all” guidelines for ART initiation are followed. HIV testing is also offered through periodic community-based outreach.
  1. **Outcomes**

The primary outcome compares between arms the proportion of self-reported lifetime HIV testing in adolescents (15-19 years).

Secondary outcomes compare between arms:

- Self-reported recent HIV testing (in last three months) in men.
- Self-reported recent HIV testing (in last three months) in older adults (≥40 years).
- Cumulative incidence of population-level ART initiation across six months^[[1]](#footnote-1)^.
- Knowledge of preventive benefits of HIV treatment.
- HIV-testing stigma.

Exploratory analyses compare between arms:

- Mutual knowledge of HIV status between sexual partners.
- Self-reported recent HIV testing (in last three months) in adolescents.
- Self-reported lifetime HIV testing in (i) men, (ii) older adults, and (iii) overall.
- Self-reported recent HIV testing (in last 12 months) in (i) adolescents, (ii) men, (iii) older adults, and (iv) overall.

Outcomes are measured through post-intervention surveys and facility data collection.

1. **Methods**
   1. **Study population**

Cluster residents aged 15 years and older are eligible for the study. Group village head clusters in the catchment areas of five government primary health centres were assessed for eligibility. Out of 53 clusters, 30 were included in the study, prioritising clusters with:

- Catchment population of at least 2000 people.
- Distance of at least five kilometres away from the health facility.
- Sufficient distance and separation from boundaries of other intended clusters.
  1. **Randomisation and blinding**

Thirty group village head clusters were randomised, with restriction factors including nearest health facility, distance from health facility, catchment population, and number of villages (Table 1). From 12,540 unique combinations falling within the restriction parameters, 1,000 were drawn by computer-generated random sampling. The final allocation was selected in a public ceremony on 16 July 2018.

Because of the nature of the intervention, the study team are not blinded to the allocation status of arms. Data are managed without reference to arm allocation where possible.

**Table 1. Restriction criteria for randomisation**

| **Restriction** | | **Number of clusters** | **Criteria** |
| --- | --- | --- | --- |
| Health facility | Chilipa | 8 | 3-5/arm |
|  | Chilonga | 5 | 2-3/arm |
|  | Makanjira | 8 | 3-5/arm |
|  | Mkumba | 5 | 2-3/arm |
|  | Phirilongwe | 4 | 2/arm |
| Number of villages | 1-5 | 15 | 6-9/arm |
|  | 6-11 | 15 | 6-9/arm |
| Population size | | Keep if average population size/arm is with ±2SD of mean | |
| Distance | | Keep if average population size/arm is with ±2SD of mean | |

- 1. **Sample size**

Sample size calculations are detailed in Table 2.

**Table 2. Sample size calculations**

**Table 2a. Lifetime testing in adolescents**

| **SOC** | **% increase, absolute** | **CL-HIVST** | **Cluster size** | **k** | **No. of clusters per arm** | |
| --- | --- | --- | --- | --- | --- | --- |
|  |  |  |  |  | **80% power** | **90% power** |
| 35.0% | 20% | 55.0% | 50 | 0.25 | 8.08 | 10.47 |
| 40.0% | 20% | 60.0% | 50 | 0.25 | 9.26 | 12.06 |
| 45.0% | 20% | 65.0% | 50 | 0.25 | 10.53 | 13.76 |
| 50.0% | 20% | 70.0% | 50 | 0.25 | 11.88 | 15.57 |

**Table 2b. Recent testing in older adults**

| **SOC** | **% increase, absolute** | **CL-HIVST** | **Cluster size** | **k** | **No. of clusters per arm** | |
| --- | --- | --- | --- | --- | --- | --- |
|  |  |  |  |  | **80% power** | **90% power** |
| 25.0% | 20% | 45.0% | 50 | 0.25 | 5.96 | 7.64 |
| 30.0% | 20% | 50.0% | 50 | 0.25 | 6.97 | 9.00 |
| 35.0% | 20% | 55.0% | 50 | 0.25 | 8.08 | 10.47 |
| 40.0% | 20% | 60.0% | 50 | 0.25 | 9.26 | 12.06 |

**Table 2c. Cumulative incidence of ART initiation**

| **SOC** | **% increase, relative** | **CL-HIVST** | **Cluster size** | **k** | **No. of clusters per arm** | |
| --- | --- | --- | --- | --- | --- | --- |
|  |  |  |  |  | **80% power** | **90% power** |
| 0.5% | 1.40 | 0.7% | 4000 | 0.25 | 15.93 | 20.98 |
| 1.0% | 1.40 | 1.4% | 4000 | 0.25 | 12.98 | 17.04 |
| 1.5% | 1.40 | 2.1% | 4000 | 0.25 | 12.00 | 15.73 |
| 2.0% | 1.40 | 2.8% | 4000 | 0.25 | 11.51 | 15.07 |

- 1. **Outcome measurement**

*Primary outcome – lifetime testing among adolescents*

The primary outcome is defined as the proportion of adolescents who self-report testing for HIV in their lifetime. The numerator is the count of adolescents aged 15-19 years old who report ever-testing for HIV in the post-intervention survey. The denominator is the count of adolescents with non-missing data (including don’t know and decline to answer responses).

*Secondary outcomes – recent testing among men*

The outcome is defined as the proportion of men who self-report testing for HIV in the last three months. The numerator is the count of men aged 15 years and older who report a recent test date less than four months from the interview date in the post-intervention survey. Test dates are given as month-year. If the month is unknown and the interview date is in 2019, test dates in 2018 and 2019 are counted. If the month is unknown and the interview date is 2018, test dates in 2018 are counted. If the year is unknown, test dates are not counted. The denominator is the count of men with non-missing data (including don’t know and decline to answer responses).

*Secondary outcomes – recent testing among older adults*

The outcome is defined as the proportion of older adults who self-report testing for HIV in the last three months. The numerator is the count of adults aged 40 years and older who report a recent test date less than four months from the interview date in the post-intervention survey. Test dates are given as month-year. If the month is unknown and the interview date is in 2019, test dates in 2018 and 2019 are counted. If the month is unknown and the interview date is 2018, test dates in 2018 are counted. If the year is unknown, test dates are not counted. The denominator is the count of older adults with non-missing data (including don’t know and decline to answer responses).

*Secondary outcomes – cumulative incidence of ART initiation*

The outcome is defined as the cumulative incidence of adults per 100,000 population initiating on ART across six months. The numerator is the count of adults aged 15 years and older who are resident in the study clusters and initiated on ART within 168 days of the start of the HIVST campaign in their respective groups (Table 3). The denominator is the adult population of study clusters, which is estimated using village and health facility data and the proportion of adults enumerated for the post-intervention survey.

*Secondary outcomes – knowledge of preventive benefits of HIV treatment*

The outcome is defined as the mean score for knowledge of preventive benefits of HIV treatment. The score is derived from five questions in the extended version of the post-intervention survey. Responses are given based on a five-point Likert scale and summed, with scores ranging from 5-25 (low to high knowledge). Questions were adapted from Obermeyer et al(1).

*Secondary outcomes – HIV-testing stigma*

The outcome is defined as the mean score for HIV-testing stigma. The score is derived from six questions in the extended version of the post-intervention survey. Responses are given based on a three-point Likert scale and summed, with scores ranging from 3-18 (low to high stigma). Questions were adapted from Boshamer et al(2).

- 1. **Data collection**

Implementation of the intervention and outcome evaluation is staggered by group (Table 3), with groups pragmatically organised based on location. Post-intervention surveys are timed 8-12 weeks after the start of the intervention in their respective groups. Facility data collection will continue for six months following the start of the HIVST campaign in their respective groups.

*Post-intervention survey*

In each cluster, one or two evaluation villages for the survey were randomly selected from villages that met the following criteria:

- Located within close proximity of the main village.
- Population of at least 500 people.

If evaluation villages have approximately 500 people, surveyors will interview all households. If evaluation villages have more than 500 people, surveyors will interview 150 households, starting with the house of the village head and proceeding in a clockwise spiral outward.

Inclusion criteria are:

- Aged 15 years and older.
- Resident in an eligible household.
- Able and willing to provide written consent, or assent for respondents aged 15-17 years old.

A random subset of respondents (~25%) will receive the extended version of the survey.

**Table 3. Intervention and evaluation groups**

| **Cluster** | **Health facility** | **Arm** | **HIVST timeline** | **Intervention group** | **Evaluation group** |
| --- | --- | --- | --- | --- | --- |
| Makanjira | Makanjira | CL-HIVST | 8-18 Oct | 1 | 1 |
| Mikochi | Makanjira | CL-HIVST | 8-18 Oct | 1 | 1 |
| Mpangama | Makanjira | CL-HIVST | 8-18 Oct | 1 | 2 |
| Malamia | Makanjira | SOC |  |  | 2 |
| Lukoloma | Makanjira | CL-HIVST | 22 Oct-1 Nov | 2 | 3 |
| Mtwana | Makanjira | SOC |  |  | 3 |
| Mtiule | Makanjira | CL-HIVST | 22 Oct-1 Nov | 2 | 4 |
| Njerenje | Makanjira | SOC |  |  | 4 |
| Mkumba | Mkumba | CL-HIVST | 5-15 Nov | 3 | 5 |
| Limbalire | Mkumba | SOC |  |  | 5 |
| Mgao | Mkumba | SOC |  |  | 5 |
| Jilamu | Mkumba | CL-HIVST | 5-15 Nov | 3 | 6 |
| Mkambiri | Mkumba | SOC |  |  | 6 |
| Songa 1 | Phirilongwe | CL-HIVST |  | 4 | 7 |
| Malopa 2 | Phirilongwe | SOC | 19-29 Nov |  | 7 |
| Malopa 1 | Phirilongwe | CL-HIVST | 19-29 Nov | 4 | 8 |
| Mlongoti | Phirilongwe | SOC |  |  | 8 |
| Chilonga | Chilonga | CL-HIVST | 3-13 Dec | 5 | 9 |
| Makunula | Chilonga | CL-HIVST |  | 5 | 9 |
| Kella | Chilonga | SOC | 3-13 Dec |  | 9 |
| Maloya | Chilonga | CL-HIVST | 3-13 Dec | 5 | 10 |
| Binali | Chilonga | SOC |  |  | 10 |
| Chalenga | Chilipa | CL-HIVST | 7-17 Jan | 6 | 11 |
| Jekete | Chilipa | CL-HIVST | 7-17 Jan | 6 | 11 |
| Malenga | Chilipa | SOC |  |  | 11 |
| Naunje | Chilipa | CL-HIVST | 7-17 Jan | 6 | 12 |
| Leveni | Chilipa | SOC |  |  | 12 |
| Masapi | Chilipa | SOC |  |  | 12 |
| Nikisi | Chilipa | SOC |  |  | 13 |
| Bamusi | Chilipa | SOC |  |  | 13 |

CL-HIVST, community-led HIV self-testing

*Facility data capture*

Clinic assistants will interview new ART patients presenting at health facilities serving the study population.

Inclusion criteria are:

- Aged 15 years or older.
- Residence in group village head clusters included in the trial.
- Initiating on ART.

1. **Statistical analysis**

Statistical analysis will be done on an intention-to-treat basis and use methods appropriate for cluster-randomised trials with a small number of clusters (3). Analysis will be done in Stata version 14.0.

- 1. **Trial flow diagram**

A trial flow diagram will be produced that conforms to the 2010 Consort statement as applicable to cluster-randomised trials(4). Response rates for households, individuals and adolescents from the post-intervention survey will be summarised.

- 1. **Sample characteristics**

Sample characteristics will also be compared by arm, overall and among adolescents. Household-level characteristics will include household composition and socioeconomic status. Individual-level characteristics will at minimum include sex, age, marital status, educational attainment, literacy, religion, ethnicity, residence status and health status.

- 1. **Unadjusted analysis**

The overall risk/mean for each cluster will be calculated, with each cluster given equal weight and a log transformation applied to the summary value for each cluster as appropriate. The risk/mean difference, 95% CI and p-value will be estimated using cluster risks/means and a t-test by arm. The risk ratio, 95% CI and p-value will be calculated using cluster log risks and a t-test by arm.

- 1. **Adjusted analysis**

The adjusted analysis is the primary analysis. Effect estimates will be adjusted for age and sex*, a priori*. Covariates for adolescents will also assessed for imbalances between arms. The adjusted analysis will adopt a two-stage approach(3). A regression model will be used to adjust for confounding bias at the individual level and include terms for the adjustment factors. Covariate-adjusted residuals will be obtained from the fitted model and used to calculate the adjusted risk/mean difference and risk ratio as appropriate.

- 1. **Sub-group analysis**

A sub-group analysis will assess differences in lifetime HIV testing among adolescents by sex and age group (15-17 years, 18-19 years).

- 1. **Sensitivity analysis**

A sensitivity analysis will not include test dates with unknown months in the outcome definition for

recent testing among men and recent testing among older adults.

- 1. **Missing data**

Missing data will be examined for each variable and for each cluster or individual participant. A systematic assessment of missingness will be conducted to ascertain the reason and possible mechanism for missing data by identifying the quantity of missing data and patterns within the data. Missingness will be examined by cluster and between randomised arms to assess for systematic biases.

- 1. **Process evaluation**

The following process measures will be summarised alongside the outcome evaluation:

- Number of HIVST kits distributed.
- Proportion of respondents who have heard of self-testing.
- Proportion of respondents who have ever self-tested.
- Proportion of respondents who have self-tested in the last 3 months.
- Proportion of self-testers with a positive result.
- Proportion of self-testers harmed before or after self-testing

**References**

1. Obermeyer CM, Bott S, Carrieri P, Parsons M, Pulerwitz J, Rutenberg N, et al. HIV testing, treatment and prevention: generic tools for operational research. Geneva: World Health Organization; 2009.

2. Boshamer CB, Bruce KE. A scale to measure attitudes about HIV-antibody testing: development and psychometric validation. AIDS Educ Prev. 1999;11(5):400-13.

3. Hayes RJ, Moulton LH. Cluster randomised trials. Portland, OR: Taylor & Francison Group, LLC; 2017.

4. Campbell MK, Piaggio G, Elbourne DR, Altman DG. Consort 2010 statement: extension to cluster randomised trials. 2012.

1. In the protocol, the outcome was defined as ‘cumulative incidence of population-level ART initiation and VMMC uptake across six months’. However, VMMC services in Mangochi were discontinued prior to the start of the trial, meaning assessment of VMMC uptake was not possible. [↑](#footnote-ref-1)
