# Supplementary material for: Effect of community-led delivery of HIV self-testing on HIV testing and antiretroviral therapy initiation in Malawi: A cluster-randomised trial
Source: PLoS Med. 2021 May 11;18(5):e1003608. doi: 10.1371/journal.pmed.1003608 (PMC8112698; doi:10.1371/journal.pmed.1003608)
Supplement: S2 Text — Supporting text, tables, and figures. (DOCX) [file pmed.1003608.s003.docx]

**Supporting information**

**Table of Contents**

[Text A. Question items for knowledge of HIV prevention and HIV testing stigma measures 2](#_Toc68858482)

[Text B. Methods for cost analysis 3](#_Toc68858483)

[Table A. Exploratory HIV testing outcomes by study arm 5](#_Toc68858484)

[Table B. Costs of the community-led HIV self-testing intervention 6](#_Toc68858485)

[Figure A. Cluster risks for primary and secondary HIV testing outcomes 7](#_Toc68858486)

[Figure B. Fidelity to community-led HIV self-testing intervention by sex and age group 8](#_Toc68858487)

# Text A. Question items for knowledge of HIV prevention and HIV testing stigma measures

**Knowledge of HIV prevention**

Questions were adapted and piloted from Obermeyer et al [1]. The score was derived from five questions using a five-point likert scale (strongly agree, agree, unsure, disagree, strongly disagree), with a range of 5-25 (low to high knowledge).

1. I believe that HIV treatment makes people with HIV less infectious.

2. I would feel safe having intercourse with someone who is HIV-positive as long as they are receiving HIV treatment.

3. I am less worried about HIV infection than I used to be.

4. HIV treatment makes me less anxious about having unprotected sex.

5. HIV treatment can help prevent a person with HIV from infecting a partner.

**HIV testing stigma**

Questions were adapted and piloted from Boshamer et al [2]. The score was derived from six questions using a three-point likert scale (strongly agree, somewhat agree, disagree), with a range of 3-18 (low to high stigma).

1. I would not want anyone I know to see me queuing for an HIV test.

2. My friends or family would not approve if I went for HIV testing.

3. It would be embarrassing if someone found out I tested for HIV.

4. You know there are problems in a marriage when the couple tests for HIV.

5. Everyone who tests for HIV is HIV-positive.

6. Testing for HIV means that you are immoral.

# Text B. Methods for cost analysis

The community-led HIV self-testing (HIVST) intervention was delivered by Population Services International (PSI) Malawi and the Malawi-Liverpool-Wellcome Trust Clinical Research Programme (MLW) as part of a broader package of HIVST distribution models.

Partial cost analysis of the intervention was undertaken from the provider perspective to estimate economic costs. Financial data from expenditure records were supplemented with economic data from microcosting. Expenditure analysis involved allocating each expenditure item to a cost category and activity. Microcosting involved direct observations and interviews with the study team and community volunteers in three sites, with one site selected per sub-district.

Shared costs were allocated by HIVST distribution model based on cost category. Costs are reported in 2018 US Dollars, with local costs converted using the median exchange rate during the period of analysis[3]. The costing period was September 2018 to January 2019.

Community costs were excluded from the analysis due to incomplete data collection. Research costs, including piloting to inform the intervention design, were also excluded.

*Start-up costs*

Start-up costs included costs of training and sensitisation activities and costs incurred in the month prior to the intervention start, with the majority of development costs spent during this period.

Training activities included a two-day participatory workshop with 157 community health action group members and an HIVST training with 190 community volunteers. A total of six pairs of workshops and trainings were administered in groups of two-to-three clusters. Costs associated with trainings included costs of venue hire, projector, staff per diem, participant sit-in allowances, office stationery, and food and drink. Common costs for training were allocated using the proportion of community volunteers.

Sensitisation activities included entry meetings with the district health office, five primary health centres, and 15 group village heads, with costs incurred for participant sit-in allowances and staff per diem. Shared costs for sensitisation, including production of information, education and communication materials, were allocated using the proportion of HIVST kits distributed. Other start-up costs included other costs incurred during the start-up period, including costs of personnel and vehicles.

Start-up costs were annualised over a two-year period [4] and assumed a 3% discount rate [5].

*Capital costs*

Capital costs included building and storage, equipment, and vehicle-related costs.

Building and storage costs included common costs for rent and were allocated using the proportion of expenditures incurred under other cost categories. Shared equipment costs were similarly apportioned across HIVST distribution models. Costs of backpacks were imputed for each volunteer (MWK 30,000; US$40). Vehicle costs included common costs for vehicle hire and were allocated using the proportion of miles from the central office to sites.

Capital costs, excluding costs of building or vehicle-related hire, were annualised over their useful life and assumed a 3% discount rate [5].

*Recurrent costs*

Recurrent costs included costs of personnel; supplies; test kits; vehicle operation, maintenance and transportation; building operation and maintenance; and other recurrent inputs.

Personnel costs included staff and consultant salaries, fringe, and per diem. Direct personnel included a program manager, program coordinator, training coordinators, M&E officers, field officer, and data clerks. Shared costs for direct and indirect personnel were allocated using the proportion of reported staff time stratified by salary grade, which was ascertained through a time use questionnaire. Gratuity for community health action group members and community volunteers was provided at MWK 7,000 (US$10) per volunteer.

Supplies costs included costs of t-shirts, data collection forms, and office stationery. Costs of t-shirts were imputed for each volunteer (MWK 4,000; US$5.50). Common costs for supplies were allocated using the proportion of HIVST kits distributed.

Costs of test kits were estimated based on the unit price for the OraQuick HIV Self-Test (US$2.50), including purchase, freight, and estimated wastage of 5%, and the number of kits distributed.

Recurrent vehicle costs included costs of vehicle fuel, operation, and maintenance, with common costs allocated using the proportion of miles from the central office to sites. Recurrent building costs included utilities and maintenance for office and warehouse buildings. Common costs for office-related buildings were allocated using the proportion of expenditures incurred under other cost categories, while common costs for warehouse-related buildings were allocated using the proportion of HIVST kits distributed.

Other recurrent inputs included communications, equipment repairs and maintenance, printing, postage and delivery, and miscellaneous fees. Shared costs for other recurrent inputs were allocated using the proportion of HIVST kits distributed.

# Table A. Exploratory HIV testing outcomes by study arm

|  | **Community-led HIVST** | | **SOC** | | **Risk or mean difference**  **(95% CI)** | **Adjusted risk or mean difference (95% CI)** * | **Risk ratio (95% CI)** | **Adjusted risk ratio**  **(95% CI)** * |
| --- | --- | --- | --- | --- | --- | --- | --- | --- |
|  | **n/N (%)** | **GM** | **n/N (%)** | **GM** | **p-value** | **p-value** | **p-value** | **p-value** |
| Mutual knowledge of HIV status between sexual partners † | 2051/2875 (71.3%) | 70.6% | 1665/2931 (56.8%) | 56.2% | 14.6% (8.5-20.7%) <0.001 | 14.1% (8.6-19.5%) <0.001 | 1.26 (1.14-1.39) <0.001 | 1.25 (1.14-1.37) <0.001 |
| Lifetime HIV testing |  |  |  |  |  |  |  |  |
| Adults ≥ 15 years | 3635/3960 (91.8%) | 91.8% | 3318/3920 (84.6%) | 84.5% | 7.3% (3.8-10.7%) <0.001 | 7.2% (4.0-10.5%) <0.001 | 1.09 (1.04-1.13) <0.001 | 1.09 (1.05-1.13) <0.001 |
| Adults ≥ 40 years | 1064/1166 (91.3%) | 91.0% | 907/1111 (81.6%) | 80.4% | 10.1% (4.2-15.9%) 0.001 | 10.2% (4.5-16.0%) 0.001 | 1.13 (1.05-1.22) 0.002 | 1.13 (1.05-1.22) 0.002 |
| Men | 1391/1577 (88.2%) | 88.2% | 1165/1495 (77.9%) | 77.2% | 11.0% (6.1-15.8%) <0.001 | 10.2% (5.8-14.7%) <0.001 | 1.14 (1.08-1.21) <0.001 | 1.13 (1.07-1.2) <0.001 |
| HIV testing in the last 3 months |  |  |  |  |  |  |  |  |
| Adults ≥ 15 years | 3145/3960 (79.4%) | 78.9% | 1556/3920 (39.7%) | 39.5% | 39.5% (33.3-45.8%) <0.001 | 39.5% (33.8-45.2%) <0.001 | 2.0 (1.8-2.22) <0.001 | 2.0 (1.81-2.2) <0.001 |
| Adolescents 15-19 years | 700/910 (76.9%) | 76.8% | 309/867 (35.6%) | 34.3% | 41.4% (32.8-49.9%) <0.001 | 39.9% (32.1-47.8%) <0.001 | 2.24 (1.85-2.71) <0.001 | 2.18 (1.83-2.6) <0.001 |
| HIV testing in the last 12 months |  |  |  |  |  |  |  |  |
| Adults ≥ 15 years | 3363/3960 (84.9%) | 84.7% | 2574/3920 (65.7%) | 65.4% | 19.3% (14.6-24.0%) <0.001 | 19.5% (15.0-24.0%) <0.001 | 1.3 (1.22-1.38) <0.001 | 1.3 (1.22-1.38) <0.001 |
| Adolescents 15-19 years | 737/910 (81.0%) | 80.9% | 497/867 (57.3%) | 57.1% | 22.5% (13.5-31.6%) <0.001 | 21.3% (13.2-29.5%) <0.001 | 1.42 (1.22-1.64) <0.001 | 1.39 (1.22-1.6) <0.001 |
| Adults ≥ 40 years | 940/1166 (80.6%) | 79.8% | 587/1111 (52.8%) | 51.5% | 27.7% (20.3-35.1%) <0.001 | 27.8% (20.6-35.0%) <0.001 | 1.55 (1.36-1.76) <0.001 | 1.55 (1.37-1.76) <0.001 |
| Men | 1277/1577 (81.0%) | 80.8% | 864/1495 (57.8%) | 57.1% | 23.7% (18.0-29.5%) <0.001 | 23.1% (17.8-28.4%) <0.001 | 1.42 (1.3-1.54) <0.001 | 1.4 (1.3-1.51) <0.001 |

GM, geometric mean (of cluster-level proportions); HIVST, HIV self-testing; SOC, standard of care.

* Analysis adjusted for sex, age group, literacy, religion, ethnicity and health status. Analysis among adolescents defines levels of age group as 16-17 years and 18-19 years. Analysis among adults ≥ 40 years defines levels of age group as 40-49 years and ≥ 50 years. Analysis among men adjusts for the same covariates except for sex.

† N=5806, with 5 missing values. Defined as individuals who have mutually disclosed with a current sexual partner their results from a negative test in the last 12 months or a positive test ever.

# Table B. Costs of the community-led HIV self-testing intervention

|  | **Community-led HIVST** |
| --- | --- |
| Total intervention costs (US$) | 138624 |
| **Outcomes** * |  |
| Number of HIVST kits distributed | 24316 |
| Number of HIV-positives identified | 576 |
| Number of new HIV-positives identified | 230 |
| Number of HIV-positives identified not on treatment | 296 |
| **Unit costs** |  |
| Cost per HIVST kit distributed (US$) | 5.70 |
| Cost per HIV-positive identified (US$) | 241 |
| Cost per new HIV-positive identified (US$) | 602 |
| Cost per untreated HIV-positives identified not on treatment (US$) | 468 |
| ART, antiretroviral therapy; HIVST, HIV self-testing.  * Of 2,956 self-testers in the community-led HIVST arm, 2.4% (n=70) were HIV-positive, 0.9% (n=28) were newly HIV-positive, and 1.2% (n=36) were previously diagnosed and not on treatment. | |

# Fig A. Cluster risks for primary and secondary HIV testing outcomes

ART, antiretroviral therapy; HIVST, HIV self-testing; SOC, standard of care. Comparison of cluster risks for primary and secondary outcomes by study arm, with blue circles indicating cluster risks and red triangles indicating geometric means of cluster risks.

**
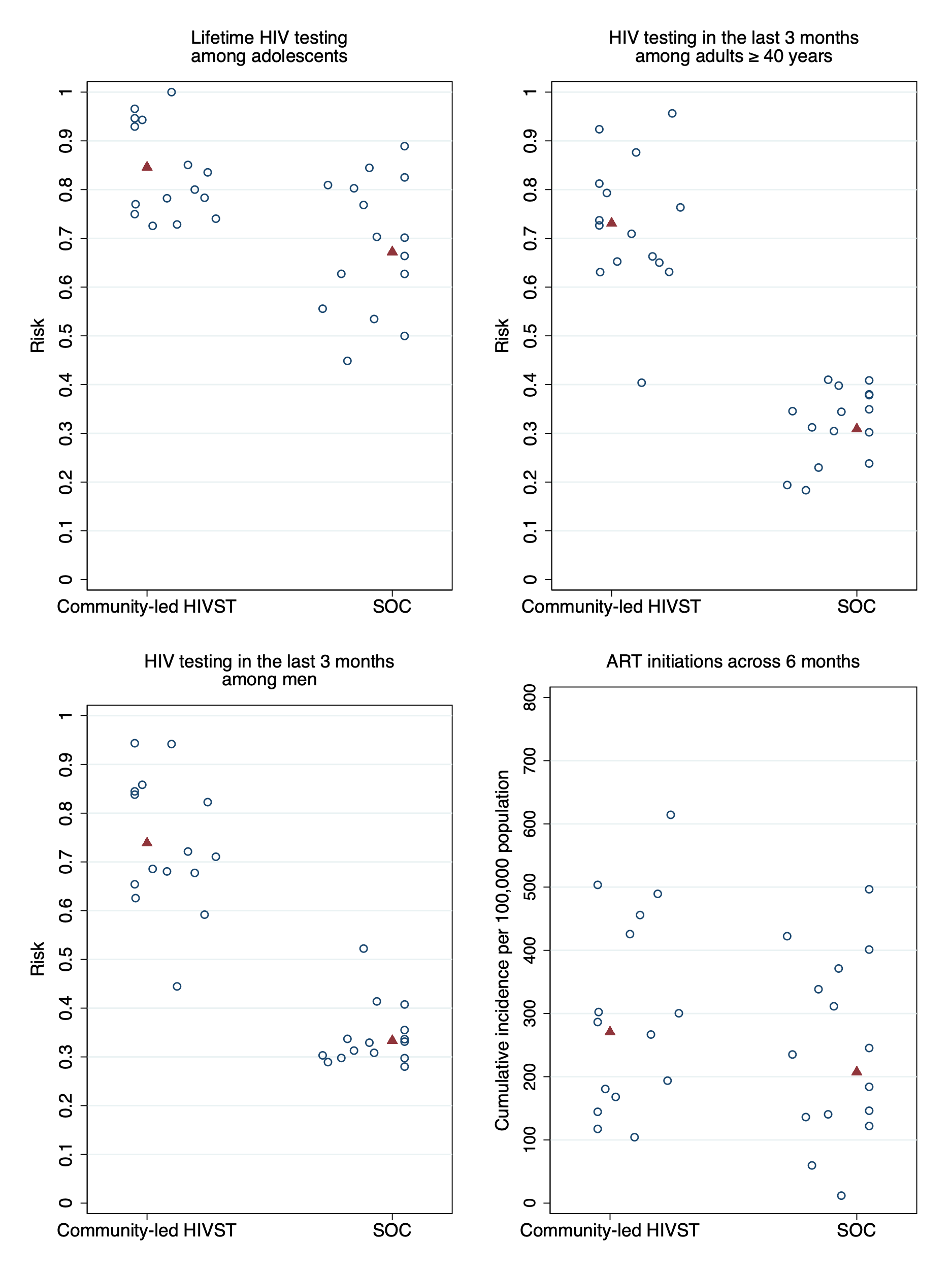
**

# Fig B. Fidelity to community-led HIV self-testing intervention by sex and age group

HIVST, HIV self-testing; SOC, standard of care. Top graphs indicate the number of HIVST kits distributed across the campaign period of the community-led HIVST intervention, with data stratified by sex and age group. Bottom graphs indicate the proportion ever self-testing and 95% CI adjusted for clustering following the community-led HIVST intervention. Data are stratified by study arm, sex and age group.


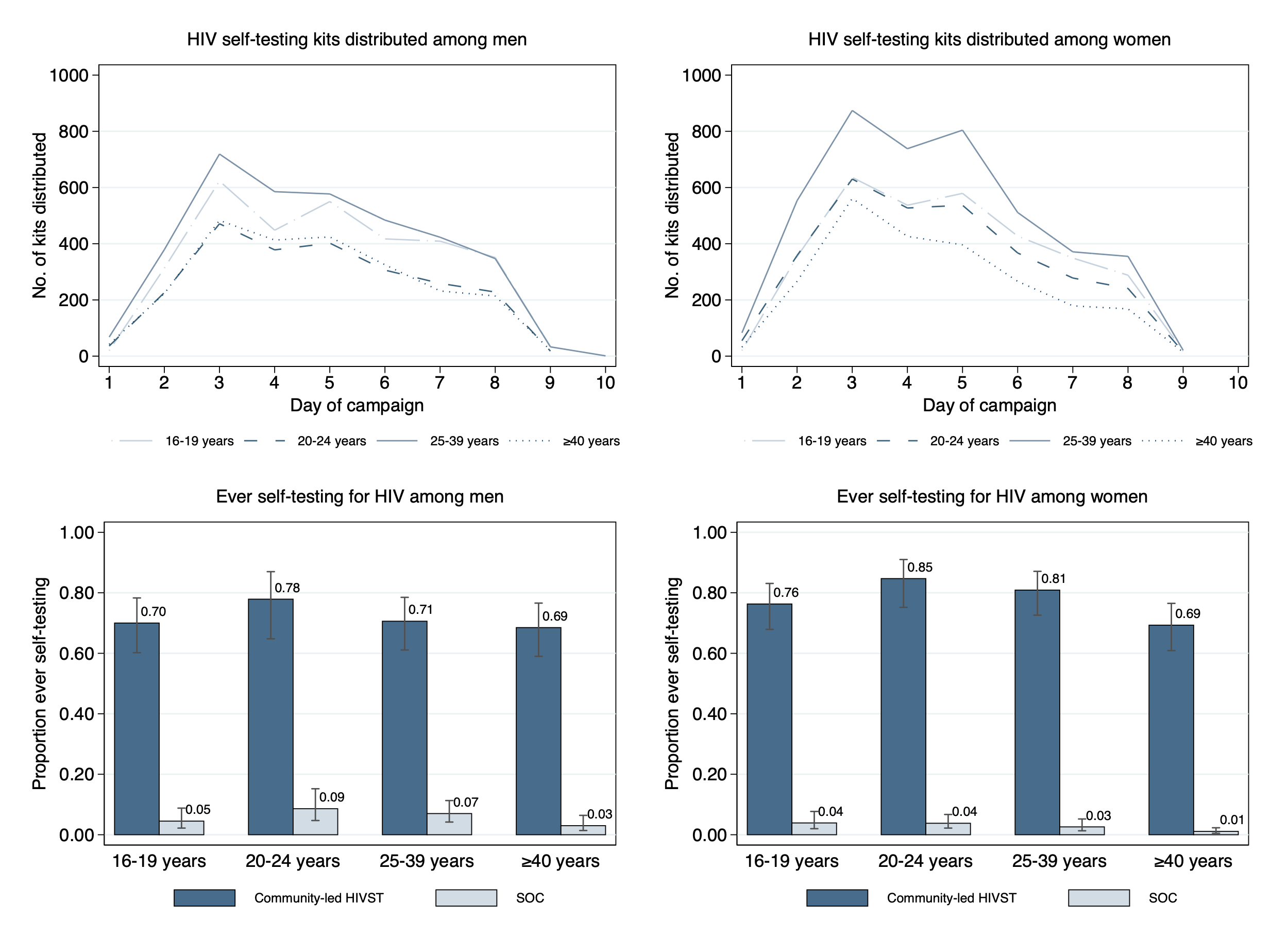


**References**

1. Obermeyer CM, Bott S, Carrieri P, Parsons M, Pulerwitz J, Rutenberg N, et al. HIV testing, treatment and prevention: generic tools for operational research. Geneva: World Health Organization, 2009.

2. Boshamer CB, Bruce KE. A scale to measure attitudes about HIV-antibody testing: development and psychometric validation. AIDS Educ Prev. 1999;11(5):400-13. Epub 1999/11/11. PubMed PMID: 10555624.

3. Bank of Malawi. Exchange Rates 2020. Available from: [https://www.rbm.mw/Statistics/MajorRates/#](https://www.rbm.mw/Statistics/MajorRates/).

4. Mangenah C, Mwenge L, Sande L, Ahmed N, d'Elbée M, Chiwawa P, et al. Economic cost analysis of door-to-door community-based distribution of HIV self-test kits in Malawi, Zambia and Zimbabwe. J Int AIDS Soc. 2019;22 Suppl 1(Suppl Suppl 1):e25255-e. doi: 10.1002/jia2.25255. PubMed PMID: 30907499.

5. Vassall A, Sweeney S, Kahn J, Gomez GB, Bollinger L, Marseille E, et al. Reference Case for Estimating the Costs of Global Health Services and Interventions: Global Health Cost Consortium; 2017. Available from: <https://ghcosting.org/pages/standards/reference_case>.
