# Supplementary material for: Effect of community-led delivery of HIV self-testing on HIV testing and antiretroviral therapy initiation in Malawi: A cluster-randomised trial
Source: PLoS Med. 2021 May 11;18(5):e1003608. doi: 10.1371/journal.pmed.1003608 (PMC8112698; doi:10.1371/journal.pmed.1003608)
Supplement: Attachment — Submitted filename: CL trial - Response to reviewers V1.2.docx [file pmed.1003608.s004.docx]

**Requests from the editors:

Please state briefly in the submission form where study data can be found - e.g., in supplementary files.** 

We have stated in the submission form that the data will be available at datacompass.lshtm.ac.uk.

**Please quote aggregate demographic details for study participants in the abstract.** 

We have added demographic characteristics for study participants to the abstract (pg. 3).

**Please add a new final sentence to the "Methods and findings" subsection of your abstract, quoting 2-3 of the study's main limitations.** 

The main limitations have been included in the abstract (pg. 4).

**At line 69, please begin the sentence with "In this study, we found that ..." or similar.**

The sentence has been modified to include the suggested phrasing (pg. 4). **In the abstract and elsewhere, please quote p values alongside 95% CI, where available.** 

We have added p-values alongside 95% CIs in the abstract and main text (pg. 4, 17, 20)

**After the abstract, we will need to ask you to add a new and accessible "author summary" section in non-identical prose. You may find it helpful to consult one or two recent research papers in PLOS Medicine to get a sense of the preferred style.** 

We have added an ‘Author summary’ section (pg. 5-6).

**Please remove the information on funding from the end of the main text. In the event of publication, this information will appear in the article metadata via entries in the submission form.** 

Information on funding has been removed from the main text (pg 28).

**Throughout the paper, please adapt reference call-outs to the following style: "... [5,6]." (i.e., no spaces within the square brackets).**

The space between reference citation numbers has been removed. **In reference 25, should that be "Taylor and Francis Group"?**

The publisher for reference 23 has been modified (pg. 30). **Please adapt the attached CONSORT checklist so that individual items are referred to by section (e.g., "Methods") and paragraph numbers rather than by line or page numbers, as the latter generally change in the event of publication. Please refer to this in the Methods section (e.g., "See S1_CONSORT_Checklist").**

We have revised the CONSORT checklist and added a reference to the checklist in the main text (pg. 8).

**Reviewer #1: 

This article describes a cluster randomized trial in Malawi of community-led delivery of HIV self-testing with a focus on impact safety and cost. The study takes on an important topic in that lack of awareness of HIV, while decreasing overall, remains high in some sub-populations and testing is the first step towards increasing testing, increasing treatment initiation, decreasing community viral suppression and eventually decreasing incidence. The study was well conducted, and the cluster randomized design was both appropriate and helps to increase the validity of the results. I commend the authors on their work and I am generally disposed toward supporting publication. Still, there are some important limitation to the work which need to be dealt with prior to this study being ready for publication.

Below are specific comments about the manuscript.

Running the intervention in conjunction with local community groups is a very nice strength of the approach and will hopefully make it more sustainable. I appreciate that the program provided support for travel for linkage to care. But is this a sustainable model? Can you talk more about this in the discussion?**

Support for linkage to routine HIV services was provided by some community health groups rather than the programme, which we have now clarified (pg. 11). The reviewer also brings up an important about sustainability, which we see as a benefit of community-led programmes and have briefly mentioned in the discussion (pg. 23).

**The number of clusters was small, so the potential for bias remains and this is worth discussing. Still, the participants were well balanced so the amount of bias is hopefully small. What is missing is the most important thing, however, which is lifetime risk of testing before the intervention period. This should be in the discussion as well.**

We have provided additional details on the sample size calculations (pg. 14), which are based on standard methods outlined by Hayes and Moulton for cluster-randomised trials [1]. Although the number of clusters was small, the trial was designed with sufficient power to measure relevant differences between arms in the primary and secondary outcomes.

Our study did not measure lifetime risk of testing through a baseline sample; we have expanded on this as a limitation in the discussion (pg. 26).

**SOC was not well described. How much testing was already being done? It would seem that the effectiveness of the intervention would be in part driven by how much people already test so this is important. Also, for the intervention arm, did facility testing continue such that the intervention is really home and facility testing? Were any other testing campaigns being done at the time.**

We have revised our description of the SOC, which included facility-based testing and periodic community-based testing under the Ministry of Health (pg. 11-12). Estimated testing coverage in the SOC arm was based on the Demographic and Health Survey 2015-16 [2], which we used to inform our sample size calculations (pg. 14). As noted by the reviewer, higher testing coverage in the SOC may have influenced the intervention effect. We have now briefly commented on this in the discussion (pg. 24).

We have also clarified that the community-led HIVST arm involved implementing the intervention in addition to the SOC (pg. 3, 8, 11-12).

**Could there have been any crossover/contamination? I suspect not, but I can't tell from the paper how close the populations were to each other and if the intervention were delivered in a way that someone could access them from outside.**

While we aimed to minimise contamination through purposive separation of cluster boundaries (pg. 9), 3.7% of participants reported HIVST use in the SOC arm (pg. 20). We have added reported spillover of HIVST as a limitation (pg. 26).

**To that point, I found details on delivery of the intervention to be lacking. You did a nice job describing the process by which the intervention was developed, but practically how were test kits delivered? They went door to door, but how often? Did they have a list of all homes to go to?**

We are currently developing a separate manuscript for the process evaluation, which will present cluster-specific implementation strategies in detail. HIVST campaigns were delivered based on strategies developed for each cluster during participatory workshops, including how, when, where, and to whom HIVST kits were to be distributed. Further, we acknowledge that these details are important and have expanded on the results for the process outcomes, including the distribution locations and distributors (pg. 22).

**It isn't clear in the methods who the study population is. Everyone in the community? Just those who wanted testing? This is a bit clearer in the results, but the idea of an evaluation population isn't made very clear in the methods. Why wasn't everyone in the cluster included in the population? I assume for cost and time reasons, but then why wasn't a random sample taken?**

We have aimed to clarify differences between the intervention and evaluation populations, as also requested by reviewer 2. The intervention population included cluster residents aged 15 years and older, who were eligible for the community-led HIVST intervention (pg. 11). This population (and the equivalent in the SOC arm) was counted in the population denominator for cumulative ART initiations (pg. 13). The evaluation population included a sample of individuals from each cluster who were eligible for the post-intervention survey (pg. 12-13).

We address the important point about the sampling strategy in the next comment.

**I have some concern about the way the evaluation population was chosen as much as I understand it. Starting with the village head household could potentially lead to those houses with more access, high probability of uptake being chosen and those households farther away which may be less likely to uptake any HIV testing being less likely to participate.**

To address the concerns raised by the reviewer, we have provided further details on the sampling frame (pg. 12-13) and also acknowledge its limitations (pg. 26).

Our sampling method was developed in response to concerns raised by the WHO Ethics Review Committee about interviewing adolescents aged 15-17 years. Specifically, they were concerned that random sampling of households, which was initially specified in the protocol, and requiring consent from household heads might expose adolescents to unintended risks, including stigma from participating in an HIV-related survey and pressure from parents or guardians to reveal their survey responses.

We therefore opted to randomly select villages that had approximately 500 residents, with all households eligible for the survey (pg. 12-13). Following cluster assessment, we discovered that nearly half of clusters only had villages with more than 500 residents. For these larger villages, we decided to use the village head household as the starting point for household enumeration, which is a common approach and would allow for a consistent sampling frame across clusters.

We recognise that the sampling approach may have led to systematic overestimation of the intervention effect, though the effect is still relatively large and our conclusions would have likely remained unchanged (pg. 26).

**The fact that outcomes are self-reported is a limitation. You mention this in the discussion but this needs more discussion. What is the likely direction and magnitude of this bias? I would think that those in the home testing group might have more incentive to say they tested due to the increased community mobilization, so it would bias away from the null.**

We have expanded on the limitations of self-reported outcomes (pg. 26). Specifically, testing outcomes were subject to misreporting due to recall or social desirability bias, including overreporting in the community-led HIVST arm following exposure to the intervention.

**In the statistical analysis, I was unclear on what this means "Risk/mean differences and
risk ratios were calculated from cluster-level summaries". Most importantly, what approach was used to adjust for the clustered design? I assume yes because you accounted for it in your sample size and using your data without cluster adjustment, I get narrower intervals, but I'm just not clear on how it was done.**

We have elaborated on the cluster-level approach for our statistical analysis, as also requested by reviewer 3 (pg. 14-15). Outcomes were analysed at cluster-level using established methods for cluster-randomised trials with a small number of clusters [1]. For the primary outcome, we calculated the proportion of adolescents who had tested in their lifetime for each cluster. These cluster-level risks were then used to calculate the risk difference and risk ratio for the intervention effect and compared between arms with a t-test. The adjusted analysis used a two-stage approach: first-stage adjusted for confounding bias at the individual-level using logistic regression and second-stage compared adjusted summary values at the cluster-level using a t-test.

**In the sample size section, and this is not really very important, but I'm curious why your control arm had much higher lifetime testing than you anticipated.**

To calculate our sample size, we used lifetime testing among adolescents as reported nationally in the Demographic and Health Survey, since we did not have district-level data [2]. However, Mangochi is a priority district for the national HIV programme and includes a high saturation of HIV services. As a result, the proportion of testing was higher than the proportion observed nationally. We have elaborated on this in the sample size (pg. 14) and discussion (pg. 24) sections.

**In the sample size you specified a 20% difference. Was this the meaningful difference? I ask because you didn't meet this even though you did find a significant result.**

We specified a 20% difference in lifetime testing among adolescents based on our previous experience with a cluster-randomised trial of community-based HIVST [3], which we have added as a reference (pg. 14). While we achieved a slightly lower effect size, we had assumed a lower proportion of lifetime testing in the SOC arm for the sample size calculations (35-50%) than the proportion observed (67%), and briefly noted this in the discussion (pg. 24).

***** Reviewer #2: 

Thank you for the opportunity review this manuscript on the effect of community-led delivery of HIV self-testing on case finding and ART initiation in Malawi. While some countries are approaching epidemic control, others are still lagging, and we must work in those countries to identify undiagnosed PLHIV. Particularly in the time of COVID-19, HIV self-testing represents a unique opportunity to provide a wide coverage of testing services to those who are unable to access testing sites due to stigma, transportation cost, lock-down restrictions, lack of awareness, etc. This manuscript provides important results indicating the efficacy of community HIV self-testing in Malawi, and will be important for moving case identification strategies forward.

Minor Revisions:

Methods: The inclusion/exclusion criteria is not entirely clear. Please better define how participants were selected and what the inclusion/exclusion criteria were for each study arm.**

We have elaborated on the inclusion criteria for clusters, the intervention population (i.e. individuals within clusters), and the evaluation population (i.e. sampled individuals within clusters). The intervention population included cluster residents aged 15 years and older, who were eligible for the community-led HIVST intervention (pg. 11). This population (and the equivalent in the SOC arm) was counted in the population denominator for cumulative ART initiations (pg. 13). The evaluation population included a sample of individuals from each cluster who were eligible for the post-intervention survey (pg. 12-13).

**Was counseling or social support provided to those who participated in HIV self-testing? 
Linkage to ART services for those who tested positive in SOC was mentioned, but was linkage to ART services also provided to those participating in HIV self-testing?**

Community volunteers provided results-based information on linkage to HIV services after self-testing, which we have now included in the description of the intervention (pg. 11). We have also clarified that community health groups were tasked with planning strategies to support linkage to care, including through phone referrals and donated transportation funds (pg. 11).

**Statistical analysis: An alpha value was not denoted in the statistical analysis section, and therefore it is unclear what level of significance is being tested.**

We have added the alpha value to the sample size calculations (pg. 14).

**Discussion: In reference to the current atmosphere with COVID-19, the use of HIV self-testing as a way to limit clinic visits, reduce HRH testing demands, and reduce in-person testing services could be added to strengthen the argument for community-led HIV self-testing implementation and expansion.**

Thank you for the suggestion. We have provided further reflection on the benefits of HIVST during the COVID-19 epidemic, including enabling ongoing provision of testing and reducing demand on healthcare workers to provide in-person testing (pg. 23-24).

***** Reviewer #3: 

This is a useful cluster-randomised trial on the effect of community-led delivery of HIV self-testing on HIV testing and antiretroviral therapy initiation in Malawi. However, there are quite a few issues needing attention.

1) Strictly speaking, The analyses are not intention to treat analyses. The definition of intention to treat analysis is 'once randomised always analysed'. However, around 10% of adolescents in each arm was missing for the primary analysis, and only 25% of those > 15 years old in each arm was available for the secondary analyses. These all happened after randomisation so could introduce potential biases. The current analyses are basically 'complete case analysis'.**

We have aimed to clarify that ‘intention-to-treat’ was used in reference to the treatment status of clusters (pg. 14). That is, participants within clusters were analysed based on cluster assignment to study arms, since clusters were randomised and the intervention was delivered at cluster-level [1]. We did not restrict our analysis based on individual-level exposure to the intervention (i.e. heard of HIVST or offered an HIVST kit).

Further, we have included as a limitation the low response rate among men, with almost one-fifth of eligible men not found (pg. 26).

**2) The primary outcome is the lifetime HIV testing among adolescents (15-19 years) self-testing vs standard care. However, it's confusing on how this short-term intervention (7 days) over a period less than a year was related to lifetime HIV testing? Because it's more or less a cross-section survey on HIV testing. There was neither lifetime follow-up nor prior information before the trial on the testing. The second outcome on short-term measure might be more related to the effect of the intervention, but lifetime testing? Can authors justify the appropriateness of the primary outcome.**

Thank you for the comment. We felt that lifetime testing was a more relevant measure for adolescents, since the need for recent testing among adolescents is highly variable and dependent on the onset of sexual debut and HIV-risk. We have added this as justification for the primary outcome (pg. 12). Further, we recognise the relevance of evaluating the intervention effect on shorter testing periods, with the results described in the main text (pg. 20) and presented fully in S4 Supporting Information.

**3) Sample size. Sample size was calculated on 20% efficacy in lifetime testing. Where did this 20% assumption come from? also what's justification of using coefficient of variation of 0.25 (assuming it is the intracluster correlation?)? This 0.25 seems too big and basically the sample size could not be reproduced. Detailed and clearer sample size calculation is needed with justifications of all the assumptions.**

We specified a 20% difference in lifetime testing among adolescents based on our previous experience with a cluster-randomised trial of community-based HIVST [3], which we have included as a reference (pg. 14).

As clarification, we used the coefficient of variation (k), rather than the intracluster correlation, to calculate the sample size based on standard methods outlined by Hayes and Moulton for cluster-randomised trials (pg. 14) [1]. Further, k=0.25 is often used as a rough guideline for sample size calculations [1]. In the observed data, we found that k=0.13 and rho=0.05 for the primary outcome.

**4) Statistical analysis. It said "Risk/mean differences and risk ratios were calculated from cluster-level summaries and compared between arms with t-test". This is not correct as t-test is for continuous variables. For categorical variables, need some other tests. It said "effect estimates were adjusted for sex and age group a priori and any imbalance between arms in adolescent covariates". However, can authors make it clear what exactly the stats models used for the adjusted analyses? What are the outcomes? What are the covariates?**

We have elaborated on the cluster-level approach for our statistical analysis (pg. 14-15). Outcomes were analysed at cluster-level using established methods for cluster-randomised trials with a small number of clusters [1]. Specifically, risk differences, mean differences, and risk ratios were calculated from cluster-level risks, means, and log risks, respectively. Cluster-level summaries were then compared between arms with a t-test. Using a two-stage approach, effect estimates were adjusted for sex and age group *a priori* and any imbalance between arms in adolescent covariates. To calculate the risk difference and risk ratio, the first stage used logistic regression to adjust for confounding bias at the individual-level. Predicted risks from this model were then summed at cluster-level and used to calculate the difference and ratio of observed and predicted values. A log transformation was applied to summaries as appropriate. The second stage used a t-test to compare covariate-adjusted summary values between arms. The same procedures were applied to calculate the mean difference, with linear regression used in the first stage.

We demonstrate this process for the risk difference in lifetime testing for adolescents. For the unadjusted analysis, we calculated the proportion of adolescents who had tested for each cluster. We then used a t-test to compare the mean of 15 cluster-level risks in the intervention arm with the mean of 15 cluster-level risks in the control arm. For the adjusted analysis, we fitted a logistic regression at the individual-level and included sex, age, and covariates that showed imbalance between arms, specifically literacy, religion, ethnicity, and self-reported health status. From this model, we calculated the observed and expected proportion of adolescents who had tested at cluster-level, which we then used to calculate the residual. We then used a t-test to compare the mean of 15 cluster-level residuals in the intervention arm with the mean of 15 cluster-level residuals in the control arm.

**5) Study design. Although the lifetime testing for adolescents is the primary outcome, it seems the investigators were more interested in households and adult residents in each cluster when designing the trial and randomising the clusters. It's a bit confusing what's rationale of the study design. Household first or adolescents first? It looks like the sample size calculation and identification of primary outcome is a post hoc decision as there are so many focus on secondary analyses which are not even powered. As we can see, the table 1 should be on the comparison of adolescents in two arms but it becomes about households? The current table 1 can be removed to supplementary information.**

Outcomes were predefined, as specified in the protocol [4]. When defining our outcomes, we were interested in the impact of the intervention among underserved subgroups (pg. 8, 12). While our primary interest was aimed at improving lifetime testing in adolescents, the intervention would have also been important in terms of increasing testing uptake in older adults and men (pg. 12). We therefore powered the study design to measure both primary and secondary outcomes for testing (pg. 14) and used a household sampling approach to include these subgroups (pg. 12-13).

We understand that the justification for the study design can be a bit confusing and have aimed to provide further details throughout the methods section. As per the reviewer’s suggestion, we have also modified Table 1 to include comparison of adolescent characteristics (pg. 16), with comparison of characteristics for the overall sample moved to the appendix.

**6) Randomisation. It only said "restricted randomisation" but exactly by what method? stratification, minimisation or blocking? Details are needed. Also there are professional software and CTU service around for the randomisation but two study PIs did this in a way that is not really understandable by professional statisticians. It should be simpler and more straightforward than what's written in the paper.**

We have included additional information on the rationale and procedures for restricted randomisation (pg. 9-10), which is a common approach for cluster-randomised trials with a small number of clusters [1,5,6]. Restricted randomisation is used to ensure balance between arms for key factors that could influence the intervention effect and can now be done in Stata using the command ‘cvcrand’. This approach is different from stratification, minimisation, or blocking, which are common techniques in individually-randomised trials.

**References**

1.	Hayes R.J., Moulton L.H. Cluster Randomised Trials. Portland, OR: Taylor & Francis Group, LLC; 2017.

2.	National Statistical Office (NSO) [Malawi] and ICF. Malawi Demographic and Health Survey (DHS) 2015-16. Zomba, Malawi, and Rockville, Maryland, USA: NSO and ICF, 2017.

3.	Indravudh PP, Fielding K, Neuman M, Chilongosi R, Mkandawire P, Nyondo E, et al. Increasing knowledge of HIV status and demand for ART using community-based HIV self-testing in rural communities: a cluster randomised trial in Malawi. 22nd International AIDS Conference (AIDS 2018); July 23-27 2018; Amsterdam, Netherlands: International AIDS Society; 2018.

4.	Adeagbo OA, Mthiyane N, Herbst C, Mee P, Neuman M, Dreyer J, et al. Cluster randomised controlled trial to determine the effect of peer delivery HIV self-testing to support linkage to HIV prevention among young women in rural KwaZulu-Natal, South Africa: a study protocol. BMJ Open. 2019;9(12):e033435. Epub 2019/12/26. doi: 10.1136/bmjopen-2019-033435. PubMed PMID: 31874891.

5.	Moulton LH. Covariate-based constrained randomization of group-randomized trials. Clinical Trials. 2004;1(3):297-305. doi: 10.1191/1740774504cn024oa. PubMed PMID: 16279255.

6.	Sismanidis C, Moulton LH, Ayles H, Fielding K, Schaap A, Beyers N, et al. Restricted randomization of ZAMSTAR: a 2 x 2 factorial cluster randomized trial. Clin Trials. 2008;5(4):316-27. Epub 2008/08/14. doi: 10.1177/1740774508094747. PubMed PMID: 18697846.
