# Supplementary material for: Effect of community-led delivery of HIV self-testing on HIV testing and antiretroviral therapy initiation in Malawi: A cluster-randomised trial
Source: PLoS Med. 2021 May 11;18(5):e1003608. doi: 10.1371/journal.pmed.1003608 (PMC8112698; doi:10.1371/journal.pmed.1003608)
Supplement: Attachment — Submitted filename: STAR_CL trial_Response to reviewers_v1.3.docx [file pmed.1003608.s005.docx]

**Requests from the editors:

Please finalize the arrangements for data release.** 

We have deposited the data at datacompass.lshtm.ac.uk, which is awaiting final approval by the editor.

**We suggest amending the text at line 34 to "... HIV infection ... in key groups including ...".**

We have made the suggested modifications to the text at line 34. **Please revisit the cumulative incidence of ART initiation, risk difference "73.2%" quoted at line 68. This appears to be quoted as "72.7%" in table 2.** 

We have carefully checked the manuscript to resolve discrepancies between the abstract (lines 69), results (lines 434), and Table 2, with a correct figure of 72.3.

**Also at line 68, please adapt the wording to make it clear what "Differences were observed ..." refers to, perhaps by linking this to the previous sentence. Please adapt the wording if this is a post-hoc analysis. Again, please make sure that the numbers quoted can be reconciled with those at line 104 and in table 2.** 

We have clarified that the differences refer to ART initiations (lines 69-70) and that the analysis was post-hoc (lines 69). We have also checked and revised the numbers quoted at

lines 68-71, 118-120, 433-439, and Table 2.

**At line 104, we ask you to adapt the wording to "apparently increased" or similar, as we believe you are quoting a secondary outcome finding where the risk of type 1 error is elevated. Please make a similar change at line 462.**

We have made the recommended changes at lines 119 and 491.

**At line 547, we suggest using the word "contamination" rather than "spillover".** 

We have revised the wording as suggested (line 577).

**Comments from the reviewers:

*** Reviewer #1: 

Overall I am happy with the responses except for:

The number of clusters was small, so the potential for bias remains and this is worth discussing. Still, the participants were well balanced so the amount of bias is hopefully small. What is missing is the most important thing, however, which is lifetime risk of
testing before the intervention period. This should be in the discussion as well.

We have provided additional details on the sample size calculations (pg. 14), which are based on standard methods outlined by Hayes and Moulton for cluster-randomised trials [1]. Although the number of clusters was small, the trial was designed with sufficient power to measure relevant differences between arms in the primary and secondary outcomes. Our study did not measure lifetime risk of testing through a baseline sample; we have expanded on this as a limitation in the discussion (pg. 26).

My point is not about power, my point is about bias. Small numbers of clusters means that the chance of lack of exchangeable populations increases and this is an important limitation of the design that needs to be discussed.**

Thank you for the clarification. We have added in the limitations that we had a small number of clusters and aimed to minimise bias through randomisation of clusters, using restriction of factors likely to be associated with the outcome, and adjustment for imbalances between arms in individual characteristics (lines 582-585). We also acknowledge that we did not measure primary and secondary outcomes in a baseline sample and adjust for baseline HIV testing, which might contribute to bias in effect estimates.  ***** Reviewer #2: 

Thank you for the opportunity re-review this paper looking at the impact of community-led HIVST on case identification and initiation on treatment. This is an important and relevant study that shows the effectiveness of HIVST in improving case identification, treatment initiation, viral suppression, and eventually reducing incidence. The author has addressed all of my and my fellow reviewer's comments in this paper with sufficient justification, and I therefore recommend this paper be accepted for publication.

*** Reviewer #3: 

Many thanks authors for their great effort to improve the manuscript. The authors have addressed most of my concerns and comments adequately. However, there is still one remaining issue needing attention. I am still not satisfied/convinced with the answer for using the lifetime HIV testing among adolescents (15-19 years) as primary outcome of the study. I can understand it from practical point of view but couldn't understand what's research hypothesis and potential causal relationship between the short-term intervention and lifetime HIV testing, direct or indirect? any evidence (similar studies) from the existing literature? The secondary outcomes are more straightforward and making sense so it's fine.**

As further clarification, we selected lifetime HIV testing for adolescents as the primary outcome since the proportion who have never tested in this age group remains higher than in older age groups. According to the 2015-16 Demographic and Health Survey, coverage of lifetime testing was respectively 49% and 35% in adolescent girls and boys; coverage of recent testing was respectively 32% and 22% in adolescent girls and boys [1]. We therefore anticipated that the community-led HIVST intervention would be introducing HIV testing to many adolescents for the first time, with lifetime testing a more pertinent outcome than recent testing.

Our hypothesis linking the community-led HIVST intervention to lifetime testing is as follows: Adolescents are a key sub-group for HIV testing and prevention programmes because of high vulnerability to HIV, rates of new HIV infection, and undiagnosed HIV. Most HIV infection in adolescents reflects sexually-acquired infection, which is dependent on the age of sexual debut. Perinatally-acquired infection can also occur, especially in settings with high HIV prevalence. In addition to the individual and public health benefits of HIV diagnosis and treatment, it is increasingly important to link HIV-negative adolescents to HIV prevention services, with highly effective biomedical methods available (e.g. voluntary male circumcision, pre-exposure prophylaxis). However, the proportion of adolescents who have never tested remains high. Therefore, our hypothesis was that the community-led HIVST intervention would increase lifetime testing through targeted implementation of kits to a sub-group with low prior exposure to any HIV testing.

To address the reviewer’s concerns, we have included additional justification for selecting lifetime testing among adolescents as the primary outcome. Specifically, lifetime testing was a more relevant measure for adolescents, since we anticipated that a high proportion of adolescents would have never tested, with the need for testing among this age group highly variable and dependent on the onset of sexual debut and HIV-risk. We therefore hypothesised that the intervention would increase coverage of lifetime testing in a sub-group with limited previous experience of HIV testing, with a similar effect achieved on recent testing (lines 271-276).

**References**

1.	National Statistical Office (NSO) [Malawi] and ICF. Malawi Demographic and Health Survey (DHS) 2015-16. Zomba, Malawi, and Rockville, Maryland, USA: NSO and ICF, 2017.
