# Supplementary material for: Effect of community-led delivery of HIV self-testing on HIV testing and antiretroviral therapy initiation in Malawi: A cluster-randomised trial
Source: PLoS Med. 2021 May 11;18(5):e1003608. doi: 10.1371/journal.pmed.1003608 (PMC8112698; doi:10.1371/journal.pmed.1003608)
Supplement: Attachment — Submitted filename: STAR_CL trial_Response to reviewers_v1.4.docx [file pmed.1003608.s006.docx]

**Requests from the editors:**

**Please finalize the arrangements for study data deposition.** 

We have deposited the data at datacompass.lshtm.ac.uk and were granted the following URL**,** <https://doi.org/10.17037/DATA.00001971>, which will be made live prior to publication.

**At line 64, please amend the text to "the proportions ... were ...".** 

We have amended the text as requested (line 64).

**Please revisit the sentence at lines 69-70, where it seems that a few additional words would be helpful to indicate that the difference is in favour of the HIVST group.**

We have changed the text to indicate that ART initiations were higher in the community-led HIVST arm than the SOC arm (lines 69-71). **At line 71, please make that "few adverse events".**

We have amended the text as requested (line 72).

**We note that reference 16 contains some superfluous information about competing interests - please read through the reference list to ensure that this and any other material not needed is removed.** 

We have reviewed the reference list and removed extraneous information.

**Please move table S1 to the main paper and adjust the table numbering as appropriate.** 

We have moved Table S1 (now Table 1) to the main text and amended the table numbers.

**Please break up the supplementary material document into individual files, so that specific items and files are referred to in the text (e.g., "See S2_Figure").** 

We have separated the supplementary file and amended their references in the text.

**The attached published trial protocol can be removed and simply referred to as a reference.**

We have removed the attachment.
